# A Study on a Method for Detecting Surface Defects in Optical Modules Based on Information Entropy Feature Extraction

**DOI:** 10.3390/e28060700

**Published:** 2026-06-17

**Authors:** Longbing Yang, Quan Xu, Min Liao, Kang Sun, Rujie Xiang, Yanbin Duan, Haonan Xu

**Affiliations:** 1 School of Mechanical Engineering, Xihua University, Chengdu 610039, China; lonbinyang@163.com (L.Y.); sunkang@stu.xhu.edu.cn (K.S.); m18982303637@163.com (R.X.); jackkingblabla@outlook.com (H.X.); 2 School of Mechanical Engineering, Southwest Petroleum University, Chengdu 610500, China; 3 Modern Agricultural Equipment Research Institute, Xihua University, Chengdu 610039, China; liaomin@mail.xhu.edu.cn; 4 Yibin Puyi Automotive Technology, Yinbin 644000, China; dyb@pam-cd.com

**Keywords:** defect detection, image processing, information entropy, effective information gain

## Abstract

Optical modules serve as the core transmission interfaces for artificial intelligence computing networks and digital communications. In recent years, demand for these modules has experienced explosive growth. During mass production, the requirements for the accuracy of surface defect detection and noise resistance have continued to rise. Existing POL detection models are susceptible to environmental noise interference; effective defect information is easily overwhelmed by noise entropy, and these models exhibit a high false negative rate for low-contrast and minute defects. This paper proposes a traditional image processing detection scheme that incorporates information entropy constraints. All experimental samples were collected from actual industrial mass production lines. The core process includes: noise suppression during the calibration stage using an entropy-weighted Hough transform; Canny edge detection combined with local entropy filtering for contour localization; and defect fusion recognition based on Hu similarity matching and entropy difference verification. Experimental results show that, compared to traditional POL methods, the proposed approach (WOMC) achieves an average improvement of 35.77% in image clarity and approximately a 2.25-fold increase in detection rate under Gaussian and salt-and-pepper noise conditions. According to statistical analysis of the experiments, this method achieved an accuracy of 96.67%, a recall rate of 97.32%, and a false positive rate of 3.31% in defect detection. In addition, the comprehensive performance score of this detection model reached 96.99%. Moreover, it does not require the deployment of deep-learning models, has a low computing power cost, and is suitable for the detection requirements of large-scale mass production.

## 1. Introduction

With the development of machine learning technology, various solutions have emerged in the field of surface defect detection of industrial products. Compared with traditional image processing algorithms, deep neural network models have significantly improved the accuracy of defect detection [1,2]. However, the training and deployment costs of deep learning models are high, and there is a lack of interpretability in the entropy increase transfer process of defect features. Therefore, traditional image processing methods that are logically traceable and do not require pre-training still account for a considerable proportion in the defect detection scenarios of industrial products. This design mainly uses traditional image processing methods to detect stains and scratches on the surface of optical module structural components [3,4]. The core realizes defect detection through three steps: horizontal correction, contour positioning, and similarity recognition. An edge-based information entropy-weighted optimization algorithm for Hough line detection is introduced to suppress noise during the calibration phase. By combining the Canny edge detector with local entropy thresholding, low-entropy false edges are filtered out, thereby enabling precise localization of the outer contour. Finally, contour similarity is evaluated, and defects are identified by comparing the Hu index and entropy difference [5].

In existing research approaches, traditional image processing methods have established a variety of technical solutions in the field of industrial defect detection. Adaptive threshold segmentation and morphological operations have been widely used to detect defects on the surfaces of electronic components. However, they are primarily suitable for low-complexity inspections and have limited ability to filter out false edges caused by changes in lighting conditions [6]. A defect feature extraction method based on the grayscale symmetric matrix achieves an accuracy rate of 89.2% in identifying stains on metal surfaces. However, because it does not incorporate entropy optimization, it lacks sufficient robustness for low-contrast defects. In the context of optical module inspection, previous attempts have combined Hough line detection with edge extraction to locate structural components. However, these methods do not perform weighted optimization of the Hough transform parameters, resulting in significant noise after tilt correction, which fails to meet the requirements of precision detection [7]. Furthermore, most traditional methods focus solely on optimizing individual detection steps and lack a coordinated design that encompasses the entire process—including “calibration, denoising, secondary filtering, and entropy difference verification”—making it difficult to strike a balance between detection efficiency and accuracy.

The limitations of these methods are primarily manifested in the following ways: traditional image processing methods rely heavily on manually designed features and lack robustness under uneven lighting and low-contrast conditions. Existing entropy-based detection methods use entropy merely as a simple feature and lack rigorous statistical validation, resulting in inconsistent reliability. Current contour-based detection methods ignore the distribution differences between standard contours and defect contours, resulting in high false positive rates. In contrast, the innovation of this method lies in proposing a statistically validated entropy distribution criterion for defect detection. Through quantitative metrics and statistical tests, significant differences between defect contours and standard contours have been verified. We have constructed a comprehensive and robust detection framework that outperforms traditional methods in both accuracy and robustness.

At the same time, in order to obtain clear images with a higher proportion of effective information, this paper proposes the WOMC image processing solution. In defect detection, filtering and denoising are key preprocessing steps to reduce noise entropy and enhance the effective information gain. Since the fiber end face image is vulnerable to interferences such as Gaussian noise (sensor electronic noise, which increases the global information entropy disorder) and salt-and-pepper noise (dust/imaging interference, which introduces local mutation entropy); therefore, it is necessary to employ targeted filtering methods that suppress noise entropy while preserving high-entropy details of defects such as scratches, pits, and contaminants, thereby laying the groundwork for subsequent defect identification and entropy difference verification [8].

The WOMC image processing scheme is designed around entropy constraints: First, convert the image to a grayscale image using weighted average, giving priority to retaining the high-entropy components of defect features; then perform Otsu threshold segmentation to maximize the inter-class information difference between the foreground and background to reduce entropy redundancy; next, apply median filtering with a 3 × 3 kernel to eliminate the local mutation entropy caused by salt-and-pepper noise; finally, use the Canny method to detect edges. This method can maximize the retention of effective defect information while suppressing noise entropy, and can achieve a high signal-to-noise ratio under the premise of ensuring image clarity.

In optical module defect detection, entropy serves as a core tool for quantifying the “disorder of image features” and the “distinctiveness of defect regions”. During the detection process, entropy essentially measures the degree of disorder in the distribution of grayscale values and edge features within local regions of the optical module image. Defect-free regions exhibit uniform grayscale values and regular edges, resulting in low entropy values; in contrast, defect regions display disordered feature distributions, leading to entropy values significantly higher than those of the surrounding normal areas. This characteristic serves as the key basis for defect identification and entropy difference verification.

Local information entropy (derived from Shannon entropy) is commonly used in optical module defect detection to calculate entropy values for the local neighborhood of each pixel in the image [9].

Let the local neighborhood of a pixel in the optical module image be a window W, and let the set of grayscale values within the window be {*q*_1_, *q*_2_, …, *q_m_*}. and the probabilities of these gray-level values occurring are *P*(*q*_1_), *P*(*q*_2_), …, *P*(*q_m_*), then the local information entropy of this window is:

(1) $$H\left(W\right)=-\sum \left[P\left(q_{m}\right)\times \log_{2}P\left(q_{m}\right)\right]$$

(2) $$\sum P\left(q_{m}\right)=1$$

In practical detection, this definition is applied to edge images extracted by the Canny algorithm to calculate the local entropy of each edge region. Regions with low entropy values are deemed to contain invalid information and are filtered out, while regions with high entropy values are retained.

## 2. Materials and Methods

### 2.1. Product Level Calibration

In real-world optical module testing environments, images captured by optical modules are prone to interference from surface scratches and imaging noise, which introduces a large number of pseudo-line features with low information entropy. Directly applying the traditional Hough transform may result in calibration errors. Therefore, this paper employs an edge information entropy-weighted Hough transform to perform horizontal calibration on images of optical modules captured by a camera lens [10]. The Hough transform is a feature extraction technique in image processing that detects objects with specific shapes using a voting algorithm [11]. This method calculates the local maxima of the cumulative results in the parameter space to obtain a set of contours matching the specific shape as the Hough transform result [12]. The principle is as follows: For any point A(x,y) in the Cartesian coordinate system, a line passing through point A satisfies the equation.

(3) Y=Kx+b

where k is the slope and b is the y-intercept. Therefore, the family of lines passing through point A(x,y) in the X−Y plane can be represented by the equation of the line. However, the Cartesian coordinate system may result in the loss of information regarding high-entropy vertical contour features; converting from Cartesian to polar coordinates is necessary to address this issue. In the polar coordinate system, the equation of a line is expressed as

**Figure 1:**
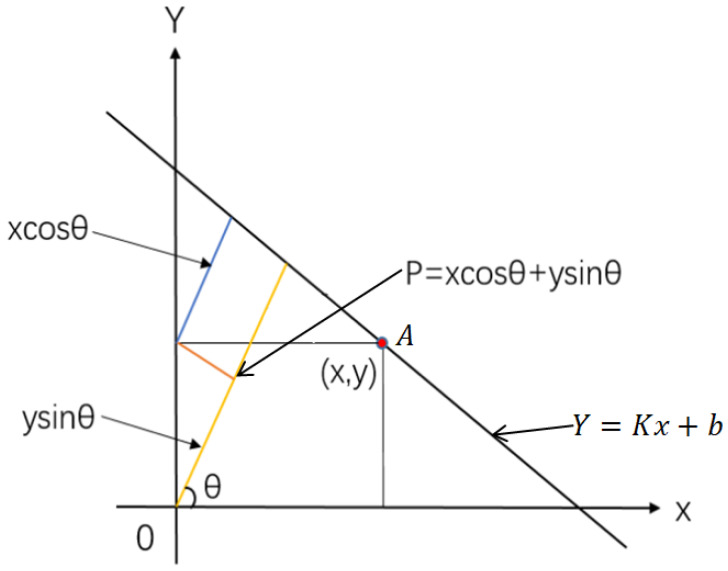
Hough Transform Coordinate System Line Chart.

**Figure 2:**
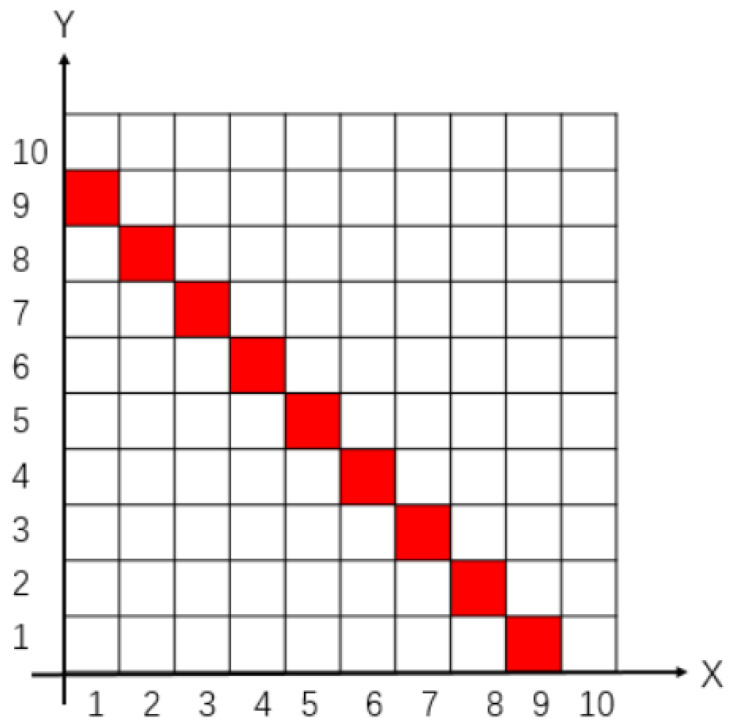
Hough Transform 10 × 10 Flat Pixel Image.

(4) p=xCosθ+ySinθ

where p is the distance from the origin to the line [13]. Figure 1 and Figure 2 illustrate a comparison of the effects of polar parameterization and entropy-weighted voting during the calibration process:

The traditional Hough transform cannot distinguish between high-entropy true contour pixels and low-entropy noise-induced false pixels, which can easily lead to interference in the voting results and result in feature bias. The entropy-weighted mechanism adopted in this paper first filters based on the entropy values of edge pixels. Only true contour pixels with local information entropy exceeding a threshold are assigned a voting weight of 1, while the voting weights of low-entropy noise and scratch-interference pixels are reduced to 0. This prevents invalid votes from interfering with the peak distribution in the parameter space. As shown in Table 1, assume there is a high-entropy straight line belonging to the optical module contour within a 10×10 planar pixel grid. All pixels on this line pass the entropy verification and receive full voting weight. Starting from the top-left pixel (1, 9), the ρ values are calculated for *θ* = 0°, 45°, 90°, 135° and 180°. The table shows that ρ corresponds to $1,5\sqrt{2},9,4\sqrt{2},and-1$, respectively, with each of these five values receiving one vote. Similarly, calculate the ρ values of the pixel point (3, 7) when *θ* = 0°, 45°, 90°, 135° and 180°, and give one vote to each of the five calculated ρ values. At this time, *p* = 5$\sqrt{2}$ has already obtained two votes. By analogy, after traversing the entire 10×10 pixel space, *p* = 5$\sqrt{2}$ will accumulate 5 votes, while the number of votes for other p-values is less than 5. Therefore, in the 10×10 pixel coordinate system, the polar coordinate equation of this straight line is:

(5) $$5\sqrt{2}=x\times \cos45{}^{\circ}+y\times \sin45{}^{\circ}$$

**Figure 3:**
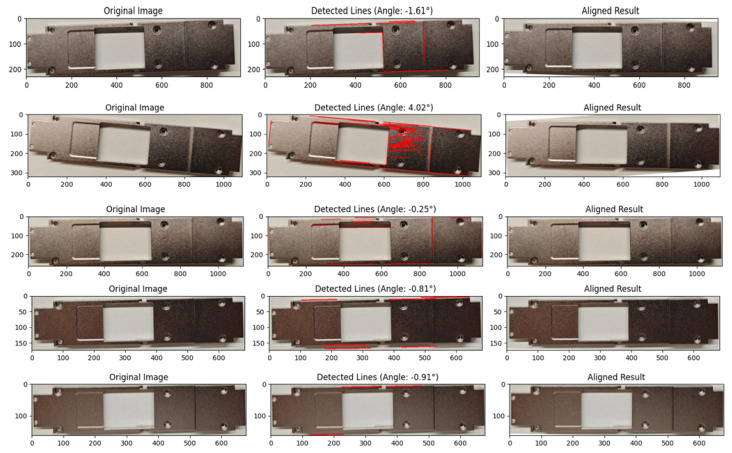
Optical Module Leveling Effect.

In practical applications, this entropy-weighted mechanism filters out more than 90% of low-entropy invalid votes, increases the peak information gain in the parameter space by more than 30%, and achieves significantly higher calibration accuracy than the traditional equally weighted Hough transform [14,15]. Figure 3 below shows a schematic diagram of the horizontal calibration of an optical module.

### 2.2. Define the Outer Contour of the Product

**Figure 4:**
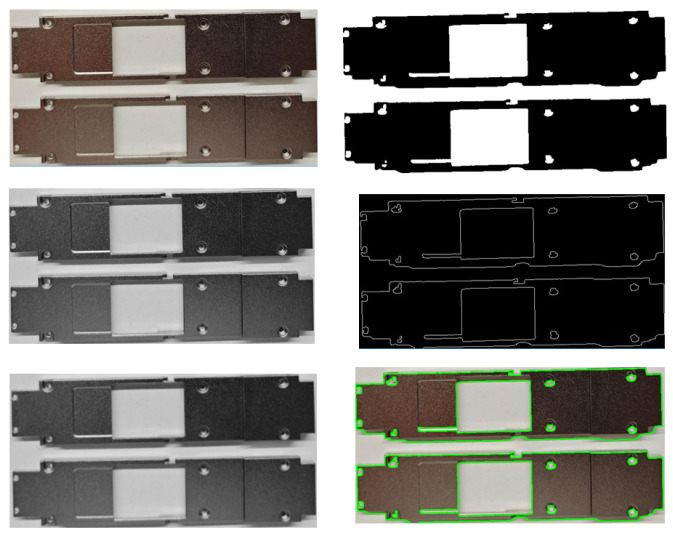
Edge Recognition for Optical Modules.

First, apply a Gaussian filter with a 5 × 5 convolution kernel to the corrected image to remove noise points. Next, perform edge detection using the Canny operator on the filtered image, followed by dilation and closure operations using a 5 × 5 identity convolution kernel [16,17,18]. Finally, as shown in Figure 4, contour detection is performed on the image. Since some noise is generated during the calibration process, an entropy weighting factor w(x,y) must be introduced during calibration to denoise the image. This paper introduces an entropy-weighted factor w(x,y) to normalize the local entropy H(x,y) of each pixel (x,y) in the image:

(6) $$w(x,y)=\frac{H_{max}-H(x,y)}{H_{max}-H_{min}}$$

Here, $H_{max}$ and $H_{min}$ represent the global maximum and minimum local entropy of the image, respectively. The larger the weight w(x,y), the less noise interference there is in the region containing that pixel, and the greater its contribution to line detection using the Hough transform.

### 2.3. Defect Detection

The defect detection of optical module products is based on the information entropy benchmark matching logic: taking the contour information entropy of the defect-free standard image as the fixed benchmark entropy, the defect is located by comparing the local entropy difference region between the image to be tested and the benchmark entropy. The core process is as follows: First, for the registered and aligned defect-free template image and the defective image to be tested, use a 3 × 3 convolution kernel for Gaussian filtering to filter out the low-entropy Gaussian noise introduced by imaging and avoid deviation of the entropy benchmark; then use a 3 × 3 unit matrix convolution kernel to perform a closing operation on the image to smooth the local entropy fluctuations and bridge the tiny breaks at the contour edges; Next, Canny edge detection is performed on the images to identify valid edge pixels with high gradient and high entropy, while filtering out invalid features in flat regions with low entropy. Finally, contour extraction is performed on both images; the resulting contour maps of the defect-free and defective images can be directly used for subsequent defect detection [19,20,21,22].

Since the optical module image only has extremely small geometric differences, such as rotation transformation and scale offset after correction, these differences will not change the global entropy distribution characteristics of the shape. Therefore, Hu moments are used as a quantitative index for contour similarity: Invariant moments are essentially a highly concentrated representation of the image shape entropy and have entropy stability for transformations such as translation, grayscale, scale, and rotation [23,24]. M.K. Hu first proposed the concept of invariant moments in 1961. In 1979, M.R. Teague proposed Zernike moments based on the orthogonal polynomial theory. Among them, Hu moments have the best entropy stability under rotation, scaling, mirroring, and translation transformations—that is, when the same or similar shapes undergo the above-mentioned transformations, their global shape information entropy distributions remain consistent, and the corresponding Hu moment values also remain basically unchanged [25]. Hu moments can be derived from the second-order and third-order central moments to form seven groups of invariant moments, as shown in the following formulas:

(7) $$M1=y_{20}+y_{02}\;$$

(8) $$M2={\left(y_{20}-y_{02}\right)}^{2}+4y_{11}^{2}\;$$

(9) $$M3={\left(y_{30}-3y_{12}\right)}^{2}+{\left(3y_{21}-y_{03}\right)}^{2}\;$$

(10) $$M4={\left(y_{30}+y_{12}\right)}^{2}+{\left(y_{21}+y_{03}\right)}^{2}$$

(11) $$\begin{array}{c} M5=\left(y_{30}-3y_{12}\right)\left(y_{30}+y_{12}\right)\left({\left(y_{30}+y_{12}\right)}^{2}-3{\left(y_{21}+y_{03}\right)}^{2}\right)\;\;\;\;\;\;\;\;\;\;\;\;\;\;\;\;\;\;\;\;\;\;\;\;\;\;\;\;\;\;\;\;\;\;\;\;\;\;\;\;\;\;\;\;\;\;\;\;\;\;\\ +\left(3y_{21}-y_{03}\right)\left(y_{21}+y_{03}\right)\left(3{\left(y_{30}+y_{12}\right)}^{2}-{\left(y_{21}+y_{03}\right)}^{2}\right) \end{array}$$

(12) $$M6=\left(y_{20}-y_{02}\right)\left({\left(y_{30}+y_{12}\right)}^{2}-{\left(y_{21}+y_{03}\right)}^{2}\right)+4y_{11}\left(y_{30}+y_{12}\right)\left(y_{21}+y_{03}\right)$$

(13) $$\begin{array}{c} M5=M7=\left(3y_{21}-y_{03}\right)\left(y_{30}+y_{12}\right)\left({\left(y_{30}+y_{12}\right)}^{2}-3{\left(y_{21}+y_{03}\right)}^{2}\right)\;\;\;\;\;\;\;\;\;\;\;\;\;\;\;\;\;\;\;\;\;\;\;\;\;\;\;\;\;\;\;\;\;\;\;\;\;\;\;\;\\ -\left(y_{30}-3y_{12}\right)\left(y_{21}+y_{03}\right)\left(3{\left(y_{30}+y_{12}\right)}^{2}-{\left(y_{21}+y_{832}\right)}^{2}\right) \end{array}$$

The Hu moment distance adopted in this paper is essentially a quantitative representation of the entropy difference in the shape information of two contours. The specific implementation method is as follows: First, traverse all the extracted contours of the image to be tested, compare the entropy of the standard shape of each contour with that of the corresponding area of the defect-free template, and convert it into a series of similarity values (the higher the similarity, the smaller the difference in the entropy distribution of the two contours). If the minimum value in this similarity sequence exceeds the predefined threshold, it indicates that the entropy distribution of this contour is significantly different from that of the standard contour, corresponding to an abnormal contour at the defect location [26].

**Figure 5:**
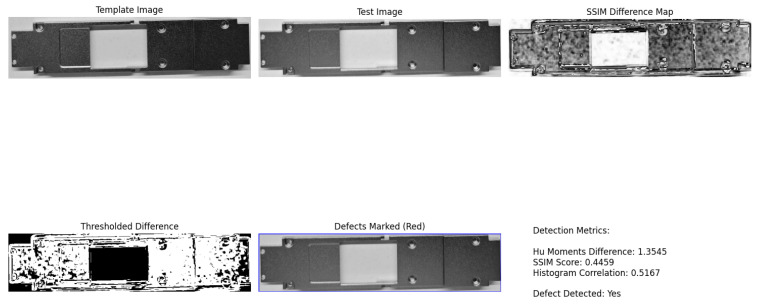
Hu Distance Defect Detection 1.

**Figure 6:**
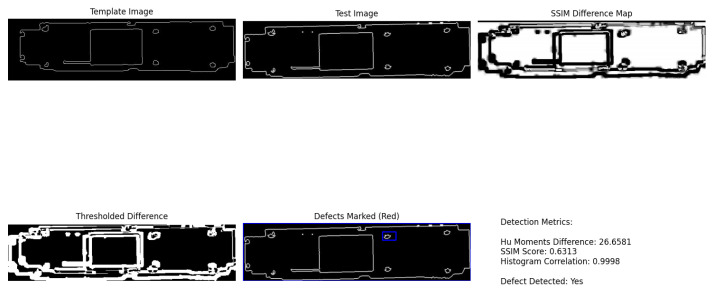
Hu Distance Defect Detection 2.

**Figure 7:**
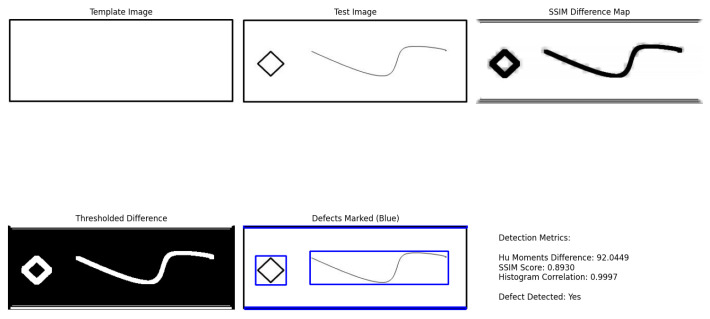
Hu Distance Defect Detection 3.

**Figure 8:**
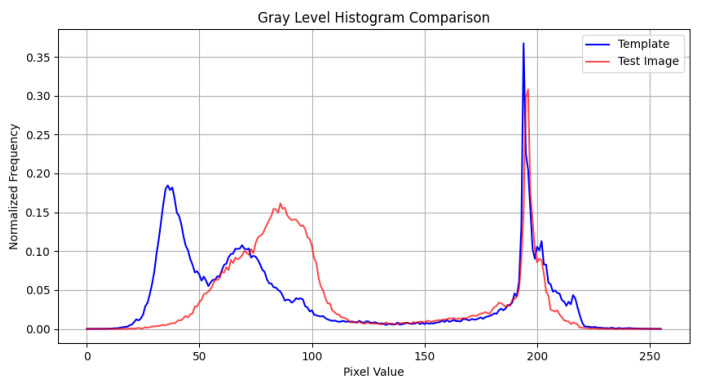
Hu Distance Defect Detection 1 Gray Scale Value.

**Figure 9:**
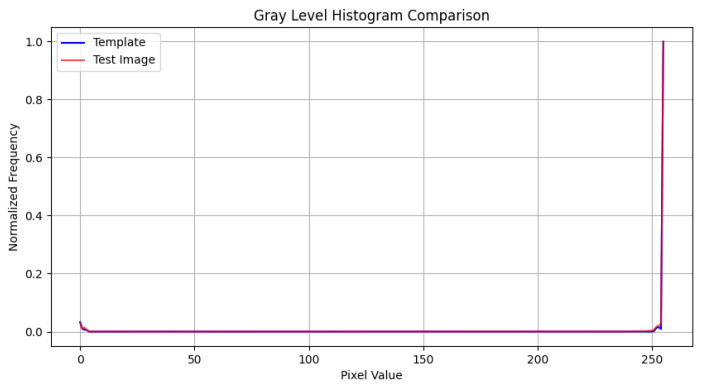
Hu Distance Defect Detection 2 Gray Scale Value.

**Figure 10:**
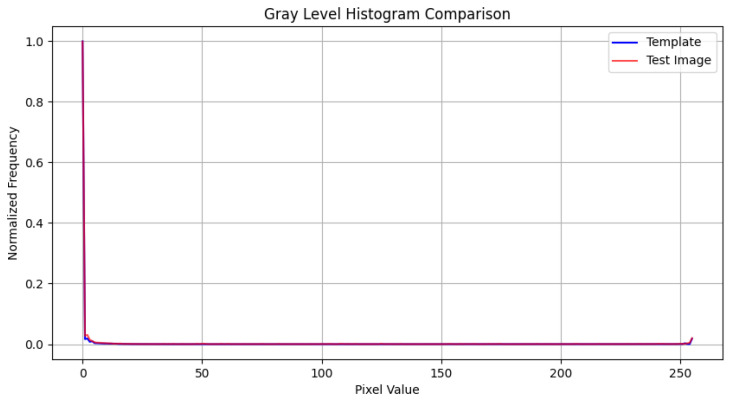
Hu Distance Defect Detection 3 Gray Scale Value.

This study employs an independent samples *t*-test to compare the entropy distributions of the defect profile and the standard (defect-free) profile. The results indicate a statistically significant difference between the two, with a *p*-value of 5.11 × 10^−9^ (see Table 2) <0.001, confirming that the entropy distributions of the defect profile and the standard profile differ significantly at a highly significant level. Subsequently, this abnormal high-entropy contour is drawn onto the image to be tested, and the effect is shown in Figure 5, Figure 6 and Figure 7; Figure 8, Figure 9 and Figure 10 depict the grayscale histogram data of the corresponding samples, which can intuitively reflect the sudden change in the global information entropy distribution brought about by the defect area.

Image sharpness is a metric that measures an image’s ability to reproduce details, reflecting the clarity of details such as object edges and textures. It is commonly quantified using metrics such as the Structural Similarity Index (SSIM) and edge intensity. A higher value indicates that the contours of defects and structural details in the image are clearer, which facilitates subsequent feature extraction. Signal-to-noise ratio (SNR) is the ratio of the intensity of the valid signal to that of the noise in an image. A higher SNR means that noise masks the valid information to a lesser extent, thereby reducing the defect misclassification rate.

**Figure 11:**
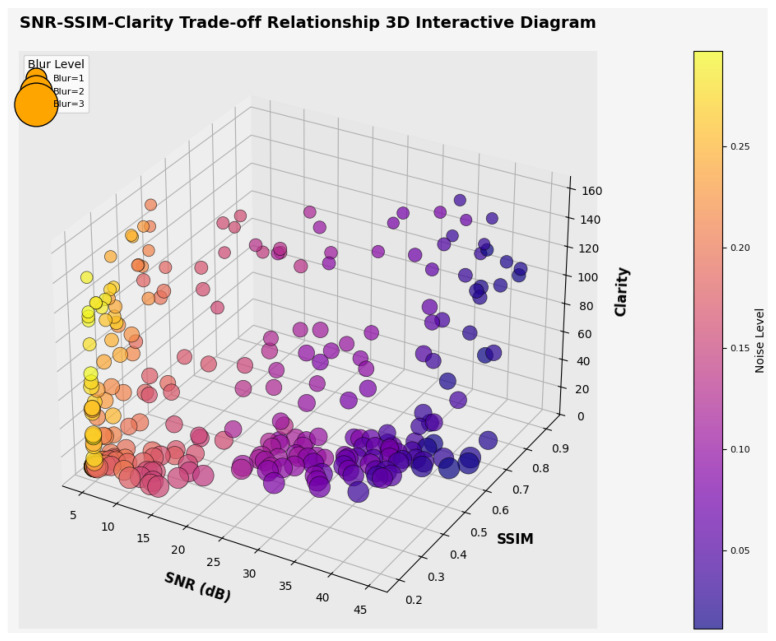
Interrelationship Diagram of Clarity, Signal-to-Noise Ratio, and Structural Similarity in Image Processing.

Finally, count the number of defect contours whose local entropy is different from the reference entropy. When the counted number exceeds the set threshold, the image is classified as a defective sample [27,28]. To more intuitively represent the internal relationship between different image quality parameters and information entropy, Figure 11 shows the interrelationships among sharpness (corresponding to the proportion of effective information entropy), signal-to-noise ratio (corresponding to the proportion of noise entropy), and structural similarity (corresponding to the global entropy distribution matching degree) in image processing.

Signal-to-noise ratio (SNR) and image clarity are key metrics for verifying the effectiveness of defect detection methods, and their validity can be demonstrated from both the perspective of detection requirements and quantification logic. From the perspective of inspection requirements, industrial defect detection must first ensure that valid information in the image is distinguishable. SNR, by quantifying the ratio of signal to noise intensity, directly reflects the extractability of defect features in the image. The higher the SNR, the less noise masks defect edges and gray-level differences, thereby preventing missed or misidentified defects caused by noise. The sharpness metric, on the other hand, focuses on the fidelity of image structure, as defect detection relies on structural information such as contours and textures. Higher sharpness results in sharper contour edges and more complete structural details, providing high-fidelity input for subsequent Hu-moment matching and entropy distribution analysis, thereby reducing feature distortion caused by image blurring. Together, these metrics cover both “information distinguishability” and “structural authenticity”, serving as direct quantitative measures of a defect detection method’s performance and meeting the core requirements of industrial applications for detection accuracy and robustness. Therefore, this solution will employ SNR and clarity metrics to validate its effectiveness.

## 3. Results

Based on the above defect detection method with fused information entropy constraints, to verify the algorithm’s ability to suppress different types of noise entropy in industrial scenarios and the preservation effect of high-entropy defect features, 50 groups of optical modules were tested under two typical industrial imaging interference conditions: Gaussian noise (global disorder entropy) and salt-and-pepper noise (local mutation entropy). Clarity essentially corresponds to the proportion of high-entropy effective defect features in the image, and the signal-to-noise ratio (SNR) corresponds to the ratio of effective information entropy to noise entropy. There is a natural trade-off between the two: to achieve good image detail recognition, it is necessary to maximize the retention of high-entropy defect features to maintain high clarity while suppressing noise entropy as much as possible and ensuring a high SNR [29].

**Figure 12:**
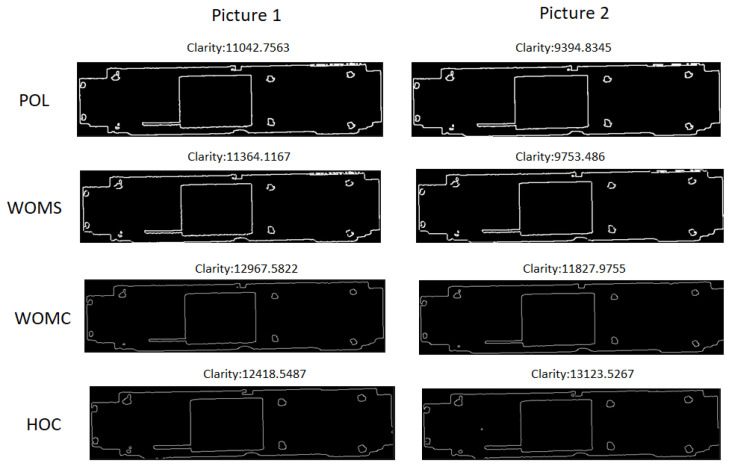
Clarity Values for Different Image Processing Methods.

**Figure 13:**
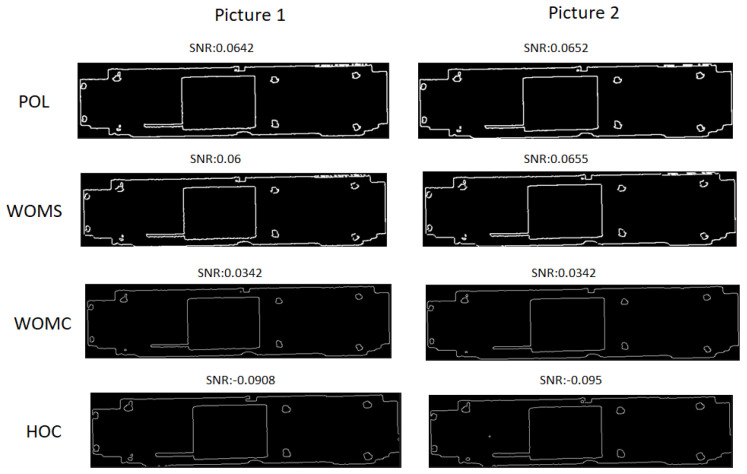
Signal-to-noise ratio values for different image processing methods.

**Figure 14:**
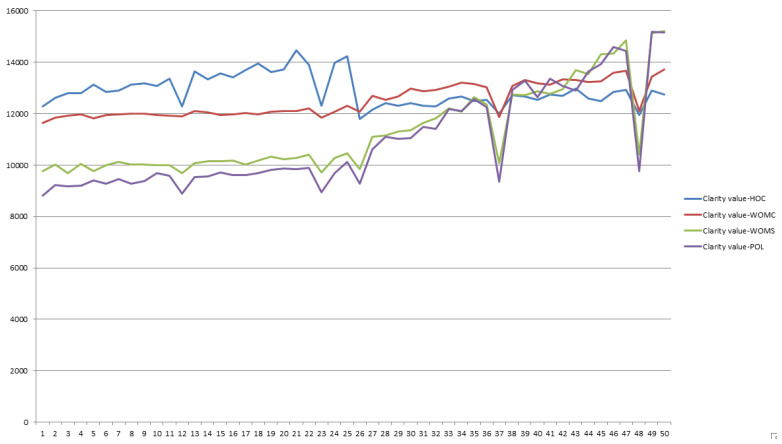
Fifty sets of image clarity values for different image processing methods.

**Figure 15:**
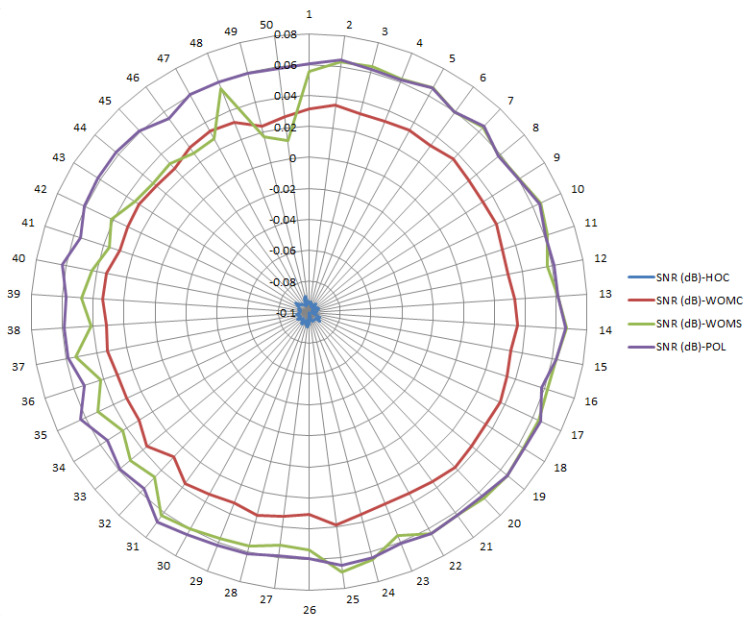
Signal-to-noise ratio values for 50 sets of different image processing methods.

In this paper, four mainstream image processing schemes were selected for comparative experiments: POL (median filtering is carried out first, followed by Otsu threshold segmentation and Laplacian edge detection. There is no entropy constraint mechanism, and it is easy to lose low-contrast high-entropy defects. WOMS (weighted average grayscale conversion, Otsu threshold segmentation, median filtering (3 × 3 kernel), and Sobel edge detection are applied in sequence. Only part of the salt-and-pepper mutation entropy can be filtered, WOMC (the fused entropy constraint scheme proposed in this paper), and HOC (grayscale conversion is carried out first, followed by Gaussian blur noise reduction, Otsu threshold method, and Canny edge detection). Gaussian filtering is likely to over-suppress high-entropy tiny defects [30,31]. The test results in Figure 12, Figure 13, Figure 14 and Figure 15 show that the WOMC image processing scheme is significantly better than the POL and WOMS methods in terms of the proportion of effective information entropy (corresponding to clarity), and is better than the HOC method in terms of the noise entropy suppression efficiency (corresponding to SNR). This method maximizes the effective information gain across various defect detection scenarios, delivering higher clarity and a better signal-to-noise ratio. Compared to existing POL detection methods, WOMC achieves an average 35.77% improvement in clarity and a 2.25-fold increase in detection speed under Gaussian and salt-and-pepper noise conditions. Furthermore, the global information entropy fluctuations in the images output by WOMC are lower, demonstrating excellent stability in terms of both clarity and signal-to-noise ratio [32,33,34].

Among the 50 sample images, samples 1–25 are primarily contaminated with Gaussian noise, while samples 26–50 are primarily contaminated with salt-and-pepper noise. As shown in the experimental statistics table, this approach demonstrates excellent consistency in suppressing Gaussian noise; however, during the suppression of salt-and-pepper noise, a very small number of noise samples exhibit significant abrupt changes. This phenomenon of abrupt changes during salt-and-pepper noise suppression is not unique to this approach; similar phenomena have been observed in other existing methods. Nevertheless, this does not detract from the clear advantages of this approach over other methods.

During the experiments, we found that when Gaussian noise and salt-and-pepper noise are too high (i.e., when the noise level exceeds 0.2), they have a significant impact on the images, making defect detection and identification impossible; salt-and-pepper noise is particularly sensitive to this. Therefore, we limited our study to drawings in environments with low levels of Gaussian and salt-and-pepper noise.

To evaluate the effectiveness of local entropy filtering in the WOMC detection scheme, this paper conducted the following comparative experiments. The effectiveness of local entropy filtering was demonstrated by applying it to the raw images from the optical module, as shown in Table 3. Additionally, its effectiveness was illustrated by comparing the signal-to-noise ratios before and after applying the WOMC detection scheme in conjunction with local entropy filtering, as shown in Table 4. As shown in Table 3, local entropy filtering reduces the signal-to-noise ratio (SNR) of the original optical module image by more than 22%. As shown in Table 4, the combination of local entropy filtering and the WOMC detection scheme improved the signal-to-noise ratio of the optical module images by 53%. These experimental results confirm that the application of local entropy filtering in the WOMC scheme further enhances the signal-to-noise ratio of the images, providing high-quality image data for subsequent defect detection.

The essence of local entropy filtering is to replace the grayscale value of the original image at point (x,y) with the entropy value of the local neighborhood of pixel (x,y) (or to combine it with the original grayscale value), thereby generating a “local entropy image”.

Calculating the Gray-Scale Probability Distribution of a Local Neighborhood $p_{\left(x,y\right)}(g)$.

For the neighborhood $W_{\left(x,y\right)}$ of pixel (x,y), we first count the frequency of all grayscale values within the window to obtain a probability distribution.

Let the total number of pixels in the window be:

(14) N=(2k+1)×(2k+1)

Let $n_{\left(x,y\right)}(g)$ be the number of pixels with a gray-scale value of g within the window. Then, the probability of a gray-scale value of g within the neighborhood $W_{\left(x,y\right)}$ is the normalized result of the frequency:

(15) $$p_{\left(x,y\right)}(g)=\frac{n_{\left(x,y\right)}(g)}{N}$$

Calculating the Entropy of a Local Neighborhood $H_{\left(x,y\right)}$.

Substituting the local probability distribution $p_{\left(x,y\right)}(g)$ obtained in Step 1 above into the Shannon entropy formula yields the “local entropy” of the neighborhood of pixel (x,y):

(16) $$H_{\left(x,y\right)}=-\sum_{g=0}^{L-1}\;p_{\left(x,y\right)}(g)\cdot {log}_{2}p_{\left(x,y\right)}(g)$$

Generating a Local Entropy Filter Image $I_{entropy}(x,y)$.

The final output of local entropy filtering is an “entropy image”, which requires mapping the local entropy values. $H_{\left(x,y\right)}$ to the image’s grayscale range (e.g., 0–255) to prevent the image from appearing too dark due to excessively low entropy values. Let the grayscale range of the original image be $\left[0,G_{max}\right]$ and the maximum possible value of the entropy is:

(17) $$H_{max}={log}_{2}L$$

Then, the normalized local entropy pixel values are:

(18) $$I_{entropy}(x,y)=\left\lfloor \frac{H_{\left(x,y\right)}}{H_{max}}\times G_{max}\right\rfloor$$

**Figure 16:**
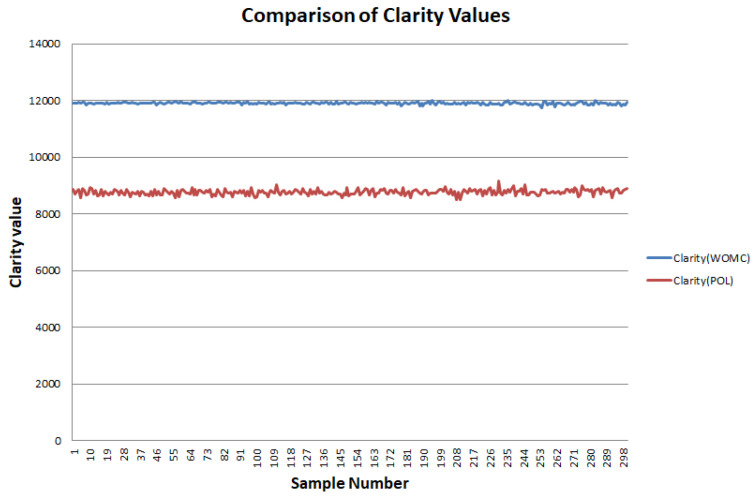
A Comparison of WOMC and POL Detection Methods in Improving Image Clarity in 300 Test Samples.

As shown in Figure 14 and Figure 15, in terms of clarity, this method (WOMC) significantly outperforms the POL detection scheme, while the HOC detection method outperforms this method in terms of signal-to-noise ratio. In this experimental detection scheme, it is necessary to achieve higher clarity values while meeting signal-to-noise ratio requirements; in this regard, this method has a clear advantage over the POL detection scheme. To further validate the reliability of this approach, the number of samples was increased to 300 for verification. As shown in Figure 16, the WOMC detection scheme performs better in terms of image clarity compared to the POL detection method. Statistical analysis reveals that the clarity values of the WOMC detection scheme are, on average, 35.77% higher than those of the POL detection scheme, with a minimum increase of 30.00% and a maximum increase of 39.64%. Meanwhile, in the 300-sample test, the WOMC detection scheme took a total of 1.087 s, with an average processing time of 3.62 ms per sample; the POL detection scheme took a total of 2.451 s, with an average processing time of 8.17 ms per sample. The WOMC detection scheme is approximately 2.25 times faster than the POL detection scheme.

The following test data is based on defect detection performed on 300 samples, of which 150 were defect-free, and the other 150 were defective. After the Hu similarity matching has preliminarily identified a defect, a secondary verification is performed by calculating the entropy difference ΔH between the region under examination and a standard defect-free region:

(19) $$\Delta H=\left|H_{test}-H_{standard}\right|\;$$

If $\Delta H>\Delta H_{th}$ ($\Delta H_{th}$ Optimize the threshold for the experiment) It is true, it is classified as a true defect; otherwise, it is a false defect (such as texture artifacts or reflections). Among 300 optical module samples, the false positive rate was 5.96% (9 false defects) without entropy difference verification; after incorporating entropy difference verification, the false positive rate dropped to 3.31% (5 false defects), effectively reducing the cost of false rejections on the mass production line and meeting the requirements of industrial applications.

According to statistical analysis, this approach achieved an accuracy rate of 96.67%, a recall rate of 97.32%, and a false positive rate of 3.31% in defect detection. Furthermore, the comprehensive performance score of the model for this detection technology reached 96.99%. The specific detection statistics are shown in Table 5 below.

(20) $$Precision=\frac{TP}{TP+FP}$$

(21) $$Recall=\frac{TP}{TP+FN}$$

(22) $$False\;detection\;rate=\frac{FP}{TN+FP}$$

Comprehensive evaluation of the model performance of the detection scheme using the *F1* score (harmonic mean):

(23) $$F1=2\times \frac{\mathrm{Precision}\times \mathrm{Recall}}{\mathrm{Precision}+\mathrm{Recall}}$$

*F1* = 2 × 0.9667 × 0.9732/(0.9667 + 0.9732) = 96.99% (24)

## 4. Conclusions

This experiment focuses on the surface defect detection task of industrial optical modules. The core objective is to extract effective defect information gain from high-noise industrial images and suppress the interference of noise entropy introduced by the environment and the imaging process. When calculating the tilt angle of an optical module using a Hough transform algorithm with edge entropy weights, the key advantage lies in incorporating the information entropy of edge regions as a weighting factor into the transformation process. Compared to the computational logic of the traditional Hough transform, which relies solely on pixel grayscale gradients, this weighting design prioritizes the retention of valid edges with high entropy values. This approach ultimately corrected the tilt angle while effectively reducing noise interference [35]. During the contour extraction stage, a strategy combining the Canny edge detector with entropy weighting was employed: first, the Canny algorithm was used to preliminarily extract edge contours, and then entropy weighting was applied to remove noise, ensuring a higher degree of alignment accuracy between the final outlined external contour and the actual boundaries of the structural components [36].

In the defect identification stage, the core of Hu moment comparison lies in the quantitative characterization of entropy differences in contour shape information; however, traditional Hu moment matching is prone to interference from noise entropy, leading to misclassification [37]. To address this issue, the WOMC detection method proposed in this paper employs a two-step optimization process: First, during the contour preprocessing stage, an entropy-based smoothing filter is introduced to adaptively smooth regions where local entropy fluctuations exceed a threshold, thereby improving the image signal-to-noise ratio; second, during the Hu moment calculation process, different regions of the contour are assigned weights based on their entropy values. Experiments demonstrate that the introduction of local entropy filtering effectively improves the signal-to-noise ratio by 53%, further enhancing the extraction of effective contour features.

The WOMC detection method can effectively suppress the entropy-increase interference caused by different noises and exhibits excellent clarity and a higher signal-to-noise ratio in defect detection under various noise environments [38]. Compared to existing POL detection methods, this approach effectively reduces the global entropy caused by Gaussian noise. In a 300-sample environment with Gaussian and salt-and-pepper noise, it achieved an average improvement of 35.77% in clarity and nearly a 2.25-fold increase in detection speed. According to statistical analysis of the experiments, this method achieved an accuracy of 96.67%, a recall rate of 97.32%, and a false positive rate of 3.31% in defect detection. In addition, the comprehensive performance score of this detection model reached 96.99%. Furthermore, the effective information entropy of the output images from this approach exhibits minimal fluctuation, demonstrating excellent stability in terms of sharpness and signal-to-noise ratio. It accurately extracts high-entropy features of surface defects on optical modules, providing robust support for practical production applications.

The experiment also revealed the areas in the existing scheme that need optimization: Firstly, the entropy difference between the entropy features of complex stain defects and the background area is small. The existing thresholds cannot effectively distinguish between them, resulting in unsatisfactory recognition effects. It is necessary to further optimize the entropy difference calculation rules to achieve accurate defect localization. Secondly, scratches belong to high-entropy linear features and will be mistakenly included in the contour line voting pool of the Hough transform, interfering with the correction of multiple detected edges and causing the correction effect of individual products to be less than fully satisfactory. In the future, an optimization plan will be developed around the hierarchical filtering logic of entropy features to continuously improve fitting consistency and robustness.

## Figures and Tables

**Table 1:** Hough Transform Pixel Coordinate Values.

-	0°	45°	90°	135°	180°	(x,y)
P1	1	5$\sqrt{2}$	9	4$\sqrt{2}$	−1	(1,9)
P2	3	5$\sqrt{2}$	7	2$\sqrt{2}$	−3	(3,7)
P3	5	5$\sqrt{2}$	5	0	−5	(5,5)
P4	7	5$\sqrt{2}$	3	−2$\sqrt{2}$	−7	(7,3)
P5	9	5$\sqrt{2}$	1	−4$\sqrt{2}$	−9	(9,1)

**Table 2:** *t*-test: Assumption of equal variances for two samples.

-	1.19 × 10^4^	8855.9
Mean	1.19 × 10^4^	8741.7925
Variance	3.20 × 10^2^	16,846.33642
Observed values	4.00	4
Pooled variance	8.58 × 10^3^	-
Hypothesized difference in means	0.00	-
df	6.00	-
t Stat	4.86 × 10	-
P (T ≤ t) (one-tailed)	2.56 × 10^−9^	-
One-tailed t-critical value	1.94	-
P (T ≤ t) (two-tailed)	5.11 × 10^−9^	-
Two-tailed t-critical value	2.45	-

**Table 3:** Changes in the signal-to-noise ratio of the original optical module image before and after local entropy filtering.

File Name	SNR Before Filtering	SNR After Filtering	Improvement (%)	Improvement (dB)
noisy_001_ratio_0.000067.png	22.23	16.53	−0.26	−5.7
noisy_001_sigma_0.000067.png	22.13	17.09	−0.23	−5.04
noisy_002_ratio_0.000133.png	22.23	16.53	−0.26	−5.7
noisy_002_sigma_0.000133.png	22.13	17.09	−0.23	−5.04
noisy_003_ratio_0.000200.png	22.2	16.53	−0.26	−5.67
noisy_003_sigma_0.000200.png	22.13	17.14	−0.23	−4.99
noisy_004_ratio_0.000267.png	22.11	16.54	−0.25	−5.57
noisy_004_sigma_0.000267.png	22.13	17.17	−0.22	−4.96
noisy_005_ratio_0.000333.png	21.88	16.52	−0.24	−5.36
noisy_005_sigma_0.000333.png	22.13	16.99	−0.23	−5.14
noisy_006_ratio_0.000400.png	22.07	16.53	−0.25	−5.54
noisy_006_sigma_0.000400.png	22.13	17.04	−0.23	−5.09
noisy_007_ratio_0.000467.png	21.62	16.54	−0.23	−5.08
noisy_007_sigma_0.000467.png	22.13	17.16	−0.22	−4.97
noisy_008_ratio_0.000533.png	22.08	16.54	−0.25	−5.54
noisy_008_sigma_0.000533.png	22.13	17.15	−0.23	−4.98
noisy_009_ratio_0.000600.png	21.89	16.52	−0.25	−5.37
noisy_009_sigma_0.000600.png	22.13	17.18	−0.22	−4.95
noisy_010_ratio_0.000667.png	22.04	16.55	−0.25	−5.49
noisy_010_sigma_0.000667.png	22.13	17.07	−0.23	−5.06
noisy_011_ratio_0.000733.png	21.73	16.53	−0.24	−5.2
noisy_011_sigma_0.000733.png	22.13	17.09	−0.23	−5.04
noisy_012_ratio_0.000800.png	21.84	16.54	−0.24	−5.3
noisy_012_sigma_0.000800.png	22.13	16.96	−0.23	−5.17
noisy_013_ratio_0.000867.png	21.36	16.56	−0.22	−4.8
noisy_013_sigma_0.000867.png	22.12	17.23	−0.22	−4.89
noisy_014_ratio_0.000933.png	21.31	16.56	−0.22	−4.75
noisy_014_sigma_0.000933.png	22.13	17.14	−0.23	−4.99
noisy_015_ratio_0.001000.png	21.72	16.54	−0.24	−5.18
noisy_015_sigma_0.001000.png	22.13	17.1	−0.23	−5.03

**Table 4:** Changes in Signal-to-Noise Ratio Before and After Local Entropy Filtering in the WOMC Testing Scheme for Optical Modules.

File Name	SNR Before Filtering	SNR After Filtering	Improvement (%)	Improvement (dB)
processed_noisy_001_ratio_0.000067.png	−14.2	−6.67	0.53	7.53
processed_noisy_001_sigma_0.000067.png	−14.2	−6.67	0.53	7.53
processed_noisy_002_ratio_0.000133.png	−14.2	−6.67	0.53	7.53
processed_noisy_002_sigma_0.000133.png	−14.2	−6.67	0.53	7.53
processed_noisy_003_ratio_0.000200.png	−14.2	−6.67	0.53	7.53
processed_noisy_003_sigma_0.000200.png	−14.2	−6.67	0.53	7.53
processed_noisy_004_ratio_0.000267.png	−14.2	−6.67	0.53	7.53
processed_noisy_004_sigma_0.000267.png	−14.2	−6.67	0.53	7.53
processed_noisy_005_ratio_0.000333.png	−14.2	−6.67	0.53	7.53
processed_noisy_005_sigma_0.000333.png	−14.2	−6.67	0.53	7.53
processed_noisy_006_ratio_0.000400.png	−14.2	−6.67	0.53	7.53
processed_noisy_006_sigma_0.000400.png	−14.2	−6.67	0.53	7.53
processed_noisy_007_ratio_0.000467.png	−14.2	−6.67	0.53	7.53
processed_noisy_007_sigma_0.000467.png	−14.2	−6.67	0.53	7.53
processed_noisy_008_ratio_0.000533.png	−14.2	−6.67	0.53	7.53
processed_noisy_008_sigma_0.000533.png	−14.2	−6.67	0.53	7.53
processed_noisy_009_ratio_0.000600.png	−14.2	−6.67	0.53	7.53
processed_noisy_009_sigma_0.000600.png	−14.2	−6.67	0.53	7.53
processed_noisy_010_ratio_0.000667.png	−14.2	−6.67	0.53	7.53
processed_noisy_010_sigma_0.000667.png	−14.2	−6.67	0.53	7.53
processed_noisy_011_ratio_0.000733.png	−14.2	−6.67	0.53	7.53
processed_noisy_011_sigma_0.000733.png	−14.2	−6.67	0.53	7.53
processed_noisy_012_ratio_0.000800.png	−14.2	−6.67	0.53	7.53
processed_noisy_012_sigma_0.000800.png	−14.2	−6.67	0.53	7.53
processed_noisy_013_ratio_0.000867.png	−14.2	−6.67	0.53	7.53
processed_noisy_013_sigma_0.000867.png	−14.2	−6.67	0.53	7.53
processed_noisy_014_ratio_0.000933.png	−14.2	−6.67	0.53	7.53
processed_noisy_014_sigma_0.000933.png	−14.2	−6.67	0.53	7.53
processed_noisy_015_ratio_0.001000.png	−14.2	−6.67	0.53	7.53
processed_noisy_015_sigma_0.001000.png	−14.2	−6.67	0.53	7.53

**Table 5:** Small-batch inspection of surface defects on modules.

Testing Methods	Sample Size for Defective Items	Sample Size for a Defect-Free Population	TP	FN	FP	TN	Precision	Recall	False Positive Rate
WOMC	150	150	145	4	5	146	96.67%	97.32%	3.31%

## Data Availability

The original contributions presented in this study are included in the article. Further inquiries can be directed to the corresponding authors.
